# Education and Income Show Heterogeneous Relationships to Lifespan Brain and Cognitive Differences Across European and US Cohorts

**DOI:** 10.1093/cercor/bhab248

**Published:** 2021-08-31

**Authors:** Kristine B Walhovd, Anders M Fjell, Yunpeng Wang, Inge K Amlien, Athanasia M Mowinckel, Ulman Lindenberger, Sandra Düzel, David Bartrés-Faz, Klaus P Ebmeier, Christian A Drevon, William F C Baaré, Paolo Ghisletta, Louise Baruël Johansen, Rogier A Kievit, Richard N Henson, Kathrine Skak Madsen, Lars Nyberg, Jennifer R Harris, Cristina Solé-Padullés, Sara Pudas, Øystein Sørensen, René Westerhausen, Enikő Zsoldos, Laura Nawijn, Torkild Hovde Lyngstad, Sana Suri, Brenda Penninx, Ole J Rogeberg, Andreas M Brandmaier

**Affiliations:** Center for Lifespan Changes in Brain and Cognition, University of Oslo, Oslo 0317, Norway; Department of Radiology and Nuclear Medicine, Oslo University Hospital, Oslo 0424, Norway; Center for Lifespan Changes in Brain and Cognition, University of Oslo, Oslo 0317, Norway; Department of Radiology and Nuclear Medicine, Oslo University Hospital, Oslo 0424, Norway; Center for Lifespan Changes in Brain and Cognition, University of Oslo, Oslo 0317, Norway; Center for Lifespan Changes in Brain and Cognition, University of Oslo, Oslo 0317, Norway; Center for Lifespan Changes in Brain and Cognition, University of Oslo, Oslo 0317, Norway; Center for Lifespan Psychology, Max Planck Institute for Human Development, Berlin 14195, Germany; Max Planck UCL Centre for Computational Psychiatry and Ageing Research, Berlin D-14195, Germany; Center for Lifespan Psychology, Max Planck Institute for Human Development, Berlin 14195, Germany; Departament de Medicina, Facultat de Medicina i Ciències de la Salut, Universitat de Barcelona, and Institut de Neurociències, Universitat de Barcelona, Barcelona 08036, Spain; Department of Psychiatry, University of Oxford, Oxford OX3 7JX, UK; Vitas AS, Oslo 0349, Norway; Department of Nutrition, Institute of Basic Medical Sciences, Faculty of Medicine, University of Oslo, Oslo 0317, Norway; Danish Research Centre for Magnetic Resonance, Centre for Functional and Diagnostic Imaging and Research, Copenhagen University Hospital - Amager and Hvidovre, Copenhagen, Denmark; Faculty of Psychology and Educational Sciences, University of Geneva, Geneva, Switzerland; UniDistance Suisse, Brig, Brig 3900, Switzerland; Swiss National Centre of Competence in Research LIVES, University of Geneva, Geneva 1212, Switzerland; Danish Research Centre for Magnetic Resonance, Centre for Functional and Diagnostic Imaging and Research, Copenhagen University Hospital - Amager and Hvidovre, Copenhagen, Denmark; Center for Neuropsychiatric Schizophrenia Research and Center for Clinical Intervention and Neuropsychiatric Schizophrenia Research, Mental Health Centre Glostrup, Glostrup 2600, Denmark; MRC Cognition and Brain Sciences Unit, University of Cambridge, Cambridge CB2 7EF, UK; Cognitive Neuroscience Department, Donders Institute for Brain, Cognition and Behavior, Radboud University Medical Center, Nijmegen 6500 GL, The Netherlands; MRC Cognition and Brain Sciences Unit, University of Cambridge, Cambridge CB2 7EF, UK; Danish Research Centre for Magnetic Resonance, Centre for Functional and Diagnostic Imaging and Research, Copenhagen University Hospital - Amager and Hvidovre, Copenhagen, Denmark; Radiography, Department of Technology, University College Copenhagen, Copenhagen 1799, Denmark; Center for Lifespan Changes in Brain and Cognition, University of Oslo, Oslo 0317, Norway; Umeå Center for Functional Brain Imaging, Umeå University, Umeå 901 87, Sweden; Department of Integrative Medical Biology, Umeå University, Umeå 901 87, Sweden; Department of Radiation Sciences, Radiology, Umeå University, 901 87 Umeå, Sweden; Division for Health Data and Digitalisation, The Norwegian Institute of Public Health, Oslo 0213, Norway; Departament de Medicina, Facultat de Medicina i Ciències de la Salut, Universitat de Barcelona, and Institut de Neurociències, Universitat de Barcelona, Barcelona 08036, Spain; Umeå Center for Functional Brain Imaging, Umeå University, Umeå 901 87, Sweden; Department of Radiation Sciences, Radiology, Umeå University, 901 87 Umeå, Sweden; Center for Lifespan Changes in Brain and Cognition, University of Oslo, Oslo 0317, Norway; Center for Lifespan Changes in Brain and Cognition, University of Oslo, Oslo 0317, Norway; Department of Psychiatry, University of Oxford, Oxford OX3 7JX, UK; Wellcome Centre for Integrative Neuroimaging, University of Oxford, Oxford OX3 7JX, UK; Department of Psychiatry, Amsterdam UMC, Vrije Universiteit, Amsterdam 1081 HJ, The Netherlands; Department of Sociology and Human Geography, Faculty of Social Sciences, University of Oslo, Oslo 0317, Norway; Department of Psychiatry, University of Oxford, Oxford OX3 7JX, UK; Wellcome Centre for Integrative Neuroimaging, University of Oxford, Oxford OX3 7JX, UK; Department of Psychiatry, Amsterdam UMC, Vrije Universiteit, Amsterdam 1081 HJ, The Netherlands; Frisch Centre, Oslo 0349, Norway; Center for Lifespan Psychology, Max Planck Institute for Human Development, Berlin 14195, Germany; Max Planck UCL Centre for Computational Psychiatry and Ageing Research, Berlin D-14195, Germany

**Keywords:** brain, cognitive function, lifespan, socioeconomic status

## Abstract

Higher socio-economic status (SES) has been proposed to have facilitating and protective effects on brain and cognition. We ask whether relationships between SES, brain volumes and cognitive ability differ across cohorts, by age and national origin. European and US cohorts covering the lifespan were studied (4–97 years, *N* = 500 000; 54 000 w/brain imaging). There was substantial heterogeneity across cohorts for all associations. Education was positively related to intracranial (ICV) and total gray matter (GM) volume. Income was related to ICV, but not GM. We did not observe reliable differences in associations as a function of age. SES was more strongly related to brain and cognition in US than European cohorts. Sample representativity varies, and this study cannot identify mechanisms underlying differences in associations across cohorts. Differences in neuroanatomical volumes partially explained SES–cognition relationships. SES was more strongly related to ICV than to GM, implying that SES–cognition relations in adulthood are less likely grounded in neuroprotective effects on GM volume in aging. The relatively stronger SES–ICV associations rather are compatible with SES–brain volume relationships being established early in life, as ICV stabilizes in childhood. The findings underscore that SES has no uniform association with, or impact on, brain and cognition.

## Introduction

Higher socio-economic status (SES), indexed by education and income, has been proposed to have facilitating and protective effects on brain and cognition (Livingston et al. 2017; Ritchie and Tucker-Drob 2018; Staff et al. 2018; Talboom et al. 2019), and has been used as a proxy for cognitive reserve over the lifespan (Jefferson et al. 2011). Positive relationships between education, income, general cognitive ability (GCA), and brain volumes have been reported in development, adulthood, and aging (Strenze 2007; Brooks et al. 2011; Noble et al. 2015; Walhovd et al. 2016; Farah 2017; Livingston et al. 2017; Ritchie and Tucker-Drob 2018; Judd et al. 2020; Lövden et al. in press). While higher SES has been seen as a dimensional facilitating or protective factor, lower SES has been indicated to confer risk to brain and cognitive function, particularly in childhood (Hanson et al. 2013; Hair et al. 2015).

SES variables are also frequently included in analyses, for example, on biological substrates of mental health, as “nuisance variables,” that is, covariates of no interest, which effects are not reported (Farah 2019). However, SES variables may not have a unified meaning or relation to brain and cognition across cohorts of varying ages and societal contexts (Tucker-Drob and Bates 2016; Ahrenfeldt et al. 2018). As indicated from the above discussion of protective and risk effects, any relationship between SES, brain and cognition may not be linear. There may well be stronger effects specific to certain ranges of SES, dependent on the context.

While higher SES has been held to be neuroprotective (Livingston et al. 2017; Ritchie and Tucker-Drob 2018; Staff et al. 2018), evidence also exists for it being neuroselective, that is, it may be a marker of other favorable traits, including genetics (Ericsson et al. 2017; Selzam et al. 2017). Both genes and environments vary with SES (Belsky et al. 2018), and any observed relationship does not need to be causal in nature. Indeed, since children inherit both genes and social class from parents, genetics linked to SES, such as education, could be spurious correlates of socially, rather than genetically transmitted advantages (Belsky et al. 2018). Differences in SES–brain–cognition associations across cohorts have implications for whether relationships can be assumed to arise from direct or indirect effects of SES in early development or aging. For instance, if education has a neuroprotective effect, then we would expect people with higher education to show less brain atrophy, and hence greater neuroanatomical volumes and also better cognitive function in aging. However, if there is a neuroselective effect of education, one might expect people with higher brain volumes and cognitive function to get more education. While a cross-sectional study such as the present cannot make fine-grained distinctions between the two, and cannot make causal interpretations, we can say something as to whether higher SES may be associated with greater neuroanatomical volumes and cognitive function, that is, a neuroprotective effect in aging, for instance. Then we would expect higher SES to be specifically related to brain volumes and cognitive function in older adulthood. On the other hand, if higher SES is associated with enhanced maturation, we would expect to see equally strong associations with childhood cognitive function, and stronger relationships to ICV, as a proxy for maximal neuroanatomical volume. More generally, different relationships across cohorts have implications for whether, when and how brain and cognitive function can be impacted by SES, or vice versa. So, while this cross-sectional multisample lifespan study cannot identify causal effects, we think the study is suited to indicate whether some causal mechanisms may be less likely to apply in general.

Here we ask whether relationships between SES, brain volumes and GCA differ significantly across cohorts—childhood/adolescence and adulthood, European or US origin—and to what extent brain variables explain SES–cognition relationships across cohorts. We address these questions by investigating how SES variables in different cohorts originating in seven European countries, as well as in the US, relate to measures of brain structure and cognitive function. We test whether age-differences (child and adolescent development vs. adulthood/aging) and differences in sample origin (within Europe and Europe vs. US) are of importance to the relationships. A further question is to what extent SES may exert influence on cognition through effects on brain structure through the lifespan, for example, either affecting brain development or aging. It should be noted that neither causality nor direction of causality is given. For instance, cognitive function could affect SES directly. People with higher cognitive ability may seek to have more education or income, and this may or may not lead to, or originate in, health behaviors that relate to brain volumes. Regardless of a possible bidirectional and complex nature, a relationship between SES and cognition may be mediated by brain characteristics. While the present data set does not lend itself to a classic mediation analysis, we analyzed partial correlations to test to what extent SES–cognition relationships change when adjusting for brain variables. Note that we do not perform this analysis to test a model of causality in terms of time-dependent effects of SES on brain and cognition. Indeed, cross-sectional analyses of longitudinal mediation are prone to bias (Maxwell and Cole 2007). However, even if SES is a distant proxy, it may be a proxy for something that affects both brain and cognition, and hence manifest in shared variance among the constructs.

The neural substrate for GCA is distributed across the brain (Fjell et al. 2015; Walhovd et al. 2016). Also anatomically widespread associations between SES and neuroanatomical features have been reported (Noble et al. 2015; McDermott et al. 2018). Hence, gross gray matter (GM) volume seems a good proxy for the brain foundations of SES–GCA relationships. GM volume is known to increase sharply along with cortical surface expansion in early childhood (Li et al. 2013), and decrease in aging along with cortical thinning and subcortical volume reductions (Storsve et al. 2014). Change in intracranial volume (ICV), on the other hand, comes to a halt after an initial period of development, and little if any age differences are seen after childhood (Pfefferbaum et al. 1994; Mills et al. 2016). ICV therefore may serve as a proxy for maximal brain size (van Loenhoud et al. 2018). Hence, if SES variables are linked to ICV, this may be seen as a relationship intrinsic to neurodevelopment. If however, SES is related to GM volume in adult and aging populations when ICV is controlled for, then this may relate to variance in brain maintenance (Nyberg et al. 2012) or neurodegeneration.

As for sample origin, one debate has centered on possibly greater effects of variation in SES in US than in Europe (Scarr-Salapatek 1971; Tucker-Drob and Bates 2016; de Zeeuw and Boomsma 2017; Figlio et al. 2017). This could be the case if the extent of stratification by SES differs between US and Europe, or if SES variation is greater in the US (Tucker-Drob and Bates 2016). Different effects of SES could also to a greater extent reflect differences in opportunity for optimal development or maintenance of brain structure and cognition in US than in Europe. For instance, differences in income could be more linked to health and education in the US where higher education and health services are not provided as part of a free or minimal-cost welfare system in contrast to some European countries. It should be noted, however, that variation in socioeconomic inequalities, educational systems, and welfare states is also substantial across birth cohorts and within Europe (Esping-Andersen Gs 1999). For instance, the UK provides a national health system, but while population health is worse in the US than in England, similar inequality in health by income have been found (Martinson 2012). Such income gradients may also apply to neurocognitive characteristics. Furthermore, the currently included cohorts are bound to vary in population representativeness, so while analyses here will illuminate differences across the specific cohorts studied, they may not readily be generalized to national or societal differences more broadly.

Finally, there is evidence that income may be more related to brain and cognition within lower income cohorts (Decker et al. 2020). While this is of interest to test, we do not have sufficient cross-cohort information to address the question of effects of poverty. Defining part of the sample as poor according to national criteria for poverty would require information of household income, size, and composition, which is not readily available for all the samples. Additionally, the criteria for poverty vary between the US and the EU and associated countries (Caminada and Martin 2011). Trying to single out individuals defined as poor in the present samples would thus be complicated and yield little power. It is difficult to set one meaningful cut-off for what may constitute lower income. Any strict division would be speculative. Hence, we chose to tentatively divide the samples by median split by income to address whether correlations with brain and cognition were significantly higher in the lower halves. In 2018, it was estimated that 1 in 6 children in the US were poor (Children's Defense Fund 2020). In view of the big US ABCD child cohort being recruited specifically to be demographically representative and such representativeness not being secured for the EU cohorts (see further discussion below), we tentatively also performed comparison analyses across US and European cohorts where the lowest 15% income participants were omitted from the US samples.

We study multiple samples within the Lifebrain consortium (Walhovd et al. 2018), and also other European and US databases with SES, brain imaging and GCA measures to which Lifebrain researchers had access, namely the UK Biobank (UKB) (Sudlow et al. 2015; Alfaro-Almagro et al. 2018), the Human Connectome Project (HCP) (Van Essen et al. 2012), and the Adolescent Brain Cognitive Development (ABCD) study (Casey et al. 2018; Garavan et al. 2018). We calculated per-site and across-site effect sizes for SES–brain–cognition relationships. The major goal of the Lifebrain consortium is to ensure a fuller exploitation, harmonization and enrichment of some of the largest longitudinal studies of age differences in brain and cognition in Europe. Hence, a stream-lined analysis of possible differences in SES–brain–cognition relationships in these data sets, in combination with other European and US databases, will serve as an assessment of the effect sizes of these relationships, and how they differ across cohorts. Such an encompassing multinational mega-analysis on SES–brain–cognition relationships across the lifespan is a novel undertaking.

Based on theoretical perspectives and evidence reviewed above, we hypothesized that SES–brain–cognition relationships would be found both in development and in adulthood/aging. We expected the relationships to vary in strength across cohorts, regardless of age. We also expected differences between US and European cohorts, but both regional differences and differences in sample characteristics within each subset may be greater than general differences between continents. Based on the variable nature of previously reported relationships, we hypothesized that SES–cognition relationships could partly be explained by differences in brain structure.

## Materials and Methods

### Samples

All research was performed under approval of relevant ethical committees/review boards, and in accordance with approved informed consent procedures. All samples were recruited to be community-dwelling participants, some were convenience samples, whereas others were contacted on the basis of populations registry information. While we do not believe development ends at a particular point, for simplicity we here use the terms “development(al)” for the child and adolescent cohorts and “adult(hood)” for the cohorts with participants 20 years of age and above. Demographics of the samples are given in Table 1, see Supplementary Material for details. For a visual representation of the age-distributions of the samples, see Supplementary Figure SS1.

**Table 1:** Overview of sample characteristics of included cohorts

Origin	Study	*N*	*M* Age	Age range	Sex M/F	*N* MRI	*N* Flu	*N* Cry	*N* Edu	*N* Inc
Norway	LCBC-Dev	767	11	4–20	0.49	767	765	767	646	374
Norway	LCBC-Adult	1148	41	20–93	0.33	1148	1125	1123	775	371
Sweden	Betula	366	63	26–97	0.48	334	364	364	363	NA
Denmark	HUBU	86	14	8–18	0.43	84	—	—	86	65
Germany	BASE-II	1828	62	24–88	0.49	414	1799	—	1590	249
Netherlands	NESDA	288	38	18–57	0.32	288	—	—	288	283
Spain	UB	305	67	36–89	0.36	299	128	226	305	—
UK	Cam-CAN	708	55	18–88	0.49	648	660	705	697	672
UK	Whitehall	780	70	60–85	0.81	755	778	779	779	635
UK	CALM	813	9	5–19	0.68	258	551	538	—	745
UK	UKB	491 261	58	38–83	0.46	39 297	184 714	—	481 610	415 914
US	ABCD	9740	10	9–11	0.52	9049	9533	9577	9723	8856
US	HCP	589	28	22–37	0.52	538	580	585	588	584
	Total	508 679	57	4–97	0.46	53 879	200 998	14 664	497 450	428 748

Fluid = measures of fluid cognitive ability, Cry = Measures of crystallized cognitive ability, Edu = measures of education, Inc = measures of income. Sex M/F refers to sex ratio, male proportion.

### Lifebrain Subsamples

The samples were derived from the European Lifebrain project (http://www.lifebrain.uio.no/) (Walhovd et al. 2018), including participants from major European brain studies: Berlin Study of Aging II (BASE II) (Bertram et al. 2014; Gerstorf et al. 2016), the BETULA project (Nilsson et al. 1997), the Centre for Attention, Learning and Memory study (CALM) (Holmes et al. 2019; Simpson-Kent et al. 2020), the Cambridge Centre for Aging and Neuroscience study (Cam-CAN) (Shafto et al. 2014), the Brain maturation in children and adolescents study (HUBU) (Madsen et al. 2018), Center for Lifebrain Changes in Brain and Cognition longitudinal studies (LCBC) (Walhovd et al. 2016; Fjell et al. 2018), the Netherlands Study of Depression and Anxiety (NESDA) (Penninx et al. 2008), the University of Barcelona brain studies (UB) (Vidal-Pineiro et al. 2014; Rajaram et al. 2016; Abellaneda-Perez et al. 2019), and the Whitehall II Imaging sub-study (WH II Imaging) (Filippini et al. 2014). In total, data from 7089 participants (5–96 years of age) were included from the Lifebrain cohorts. However, all participants and all cohorts did not contribute all categories of data, as detailed in Table 1 and Supplementary Material. Importantly, MRI-derived ICV and GM measures were available for 4995 participants from the Lifebrain cohorts.

#### UKB

The UK Biobank (UKB) recruited 502 649 participants aged 37–73 years from 2006 to 2010 (Guggenheim et al. 2015). Ethical approval was obtained from the National Health Service National Research Ethics Service (Ref 11/NW/0382) and all participants provided written informed consent. Here, the dataset released February 2020 was used, consisting of 502 507 participants, of whom 40 682 had undergone MRI scanning. After applying exclusion criteria (see Supplementary Material), 491 261 had sufficient valid information to be included in the final analyses, of whom 39 297 had a valid MRI.

#### HCP

The Human Connectome Project (HCP) is funded by the US National Institute of Health (NIH) (http://www.neuroscienceblueprint.nih.gov/connectome/). The consortium led by Washington University and the University of Minnesota (the “WU-Minn HCP Consortium”) aims to study brain connectivity and function with a genetically informative design in 1200 individuals using four MR-based modalities plus MEG and EEG. Behavioral and genetic data are also acquired from these participants. After application of exclusion criteria, 538 participants with MRI were included. For further information, see Supplementary Material.

#### ABCD

The Adolescent Brain Cognitive Development (ABCD) study aims to track human brain development from childhood through adolescence (Casey et al. 2018). ABCD has recruited >10 000 9–10-years olds across 21 US sites with harmonized measures and procedures, including imaging acquisition https://abcdstudy.org/scientists-workgroups.html and processing (Hagler Jr. et al. 2019). A goal of the ABCD study is that its sample should reflect, as best as possible, the sociodemographic variation of the US population (Garavan et al. 2018). For ABCD, the dataset release 2.0.1 was used, consisting of 11 875 participants at baseline, of whom 9740 had sufficient valid information to be included in the final analyses (9049 with MRI).

### General Procedures

We used all available Lifebrain cohorts that provided at least two of the constructs of interest: GCA (crystallized and/or fluid intelligence), SES (income and/or education), and brain structure (GM volume and ICV), resulting in a total of 10 Lifebrain studies. Of the Lifebrain studies, 9 provided measures of education, 8 of income, 9 of brain structure, and 8 of crystallized and/or fluid intelligence, used to compute measures of GCA. In addition, analyses were performed on UKB, HCP, and ABCD.

For each study, we gathered all cognitive tests that we considered measuring fluid and/or crystallized intelligence. There were multiple tests for GCA in each cohort. Using principal component analysis, we reduced GCA to its first principal component. With this approach, we could analyze the correlations of four constructs of interest that we refer to as GCA, income, education, and neuroanatomical volume. For details on how these constructs were recorded per study, see Supplementary Material. For measures of neuroanatomical volumes, we gathered FreeSurfer-based estimates of total GM volume and ICV. We meta-analyzed Spearman rank-order correlations with bootstrapped standard errors based on 1000 replications each. The bootstrapped standard errors served as weights for the meta-analysis. For GM and ICV, we ran separate regressions for each cohort predicting volume by age and checking whether absolute residuals exceeded a relatively liberal four standard-deviations criterion. If so, the respective participants were entirely deleted from the following analysis. For details, see SM.

### Magnetic Resonance Imaging Acquisition and Analysis

T1-weighted structural scans were acquired at Siemens (Erlangen, Germany), Philips, and GE scanners at the various sites. Further information on MRI scanning and processing is given in SI, and MRI sequence parameters per cohort are given in Supplementary Table SS1. Images were processed with FreeSurfer, mainly version 6.0 (https://surfer.nmr.mgh.harvard.edu/) (FreeSurfer 5.3 was used for Whitehall II, HCP and ABCD). Because FreeSurfer is almost fully automated, to avoid introducing possible site-specific biases, gross quality control measures were imposed and no manual editing was done.

### Demographic Measures

For all samples, age was measured in years and months, and converted to a three-decimal numeric value for analyses. Sex was coded as 0 for males and 1 for females. For details on how education and income was recorded, see Supplementary Material. In general, estimates of parental education and income were used for developmental samples, whereas participant income and education were recorded for adult samples.

### Cognitive Tests

For GCA, national versions of a series of batteries and tests were used, see Supplementary Material for details. These included tests from the Wechsler Abbreviated Scale of intelligence ((Wechsler 1999) LCBC, CALM), Wechsler Primary and Preschool Scale of Intelligence III ((Wechsler 2008a) LCBC – below age 6.5 years), the Wechsler Adult Intelligence Scale R/III/IV ((Wechsler 1997, 2008b) UB, Betula, Whitehall II), Wechsler Individual Achievement Test ((Wechsler 2005) CALM), Test of Premorbid Functioning ((Wechsler 2011) Whitehall II), Cattell Culture Fair ((Cattell and Cattell 1973) Cam-CAN), National Adult Reading Test ((Nelson and Willison 1991) UB), NIH toolbox ((Gershon et al. 2013) ABCD, HCP), as well as local batteries.

### Statistics

Meta-analyses were computed based on the primary outcome of a single effect size $r$, the pair-wise Spearman correlations among constructs. Spearman correlations were chosen as we did not necessarily expect relationships between SES, brain and cognition to be linear, but likely monotonic. We used pairwise complete observations to compute correlations. When constructs had more than one indicator, we used principal component analysis as dimensionality reduction techniques to obtain factor score estimates. In order to obtain PCA estimates from missing data, missing data have to be imputed. Missing data in GCA were imputed using the regularized iterative PCA algorithm (with a single component) as implemented in the R package missMDA (Josse and Husson 2016), which provides a function for estimating imputed PCA components using an iterative (expectation–maximization) algorithm. We are convinced that this type of imputation works well, since we can assume that a strong *g*-factor exists that can be leveraged by this type of algorithm. As a follow-up analysis, we generated missing data pattern matrices for several of the studies in the meta-analysis, which we provide in Supplementary Figure SS2A–J. A separate PCA was conducted per cohort, and Supplementary Table SS2 shows which measures per cohort were included in the first component, along with principal components loadings and explained variance.

All statistical tests were two-sided. Meta-analytic estimates of correlations and their precisions were obtained from the metafor package (Viechtbauer 2010). As our primary outcome of interest is the latent correlation of pairs of constructs of interest (crystallized/fluid intelligence, income, education, and neuroanatomical volumes), effect size estimates were weighted by their inverse bootstrapped standard error (which implicitly considers sample size differences among cohorts). We additionally tested the extent to which the relations between cognitive ability and SES changed when adjusting for the brain variables, by testing the difference between the GCA–SES correlations adjusted for age and sex and the same correlations additionally adjusted for ICV, GM, or both. Note that we do not do this as a classic mediation analysis with strong causal interpretation, we merely run semipartial correlations. We report mean effect size and 95% confidence interval (CI) for each study and for the meta-analytic effect size estimates. If the 95% CI did not include zero, the null hypothesis of no correlation could be rejected at a $\alpha$ level of 0.05. The meta-analysis was based on a random-effects model (Hedges and Olkin 2014), in which both the within-study variance and the between-study variance form the variance component used to calculate study-specific weights (Field 2001). In contrast to a fixed-effects model, it is often described as more conservative. Importantly, the random-effects model accounts for between-study heterogeneity, ${\tau}^2$, which itself is an outcome of interest for our analysis. To describe and test the heterogeneity in our results, we report ${I}^2$, the ratio of between-study heterogeneity, ${\tau}^2$ over observed variability (Higgins et al. 2003). ${I}^2$ can be considered a standardized effect size estimate of heterogeneity or inconsistency across studies with larger values meaning a presence of more heterogeneity. To assess whether observed heterogeneity in the estimated correlations across studies are compatible with chance alone, we also report $P$ values of Cochrane’s $Q$ test of heterogeneity.

## Results

### Regional Cortical Associations with GCA and SES

**Figure 1:**
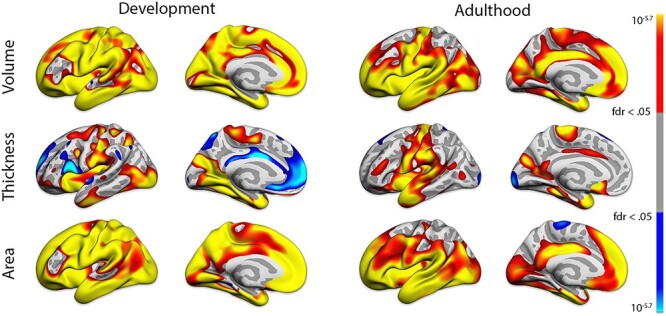
Effects of GCA on cortical volume, area and thickness. Results corrected by false discovery rate < 0.05.

**Figure 2:**
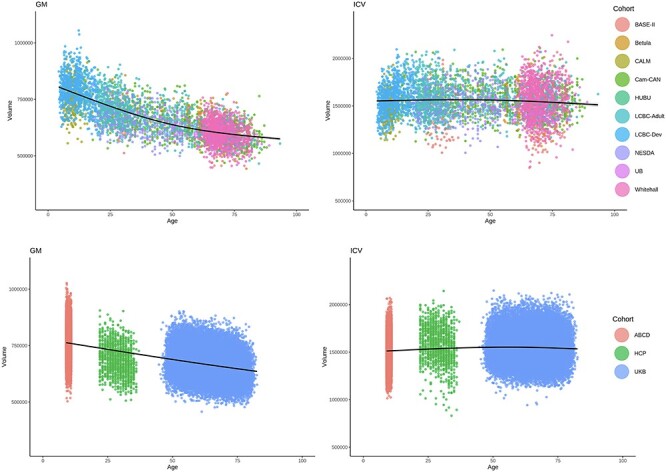
Age-related differences in GM and ICV. Individual data points are shown across upper panel: All Lifebrain cohorts (BASE-II from Germany, Betula from Sweden, CALM, Cam-Can and Whitehall from the UK, HUBU from Denmark, LCBC from Norway, NESDA from the Netherlands, and UB from Spain), and lower panel: ABCD and HCP from the USA, and UKB from the UK,. The black line represents a smoothed (3 knots) average difference. All data after outlier removal are shown.

First, we validated that global GM volume is a better measure of brain structure in relation to GCA than regional volume, thickness or area. We ran general linear models (GLM) vertex-wise across the cortical surface, with GCA as predictor, and sex, study, age, age^2^, and ICV as covariates, for the participants for whom reconstructed surfaces were available (development < 20 years, *n* = 9689; adulthood ≥ 20 years, *n* = 39 143, see Supplementary Material for details). This analysis (Fig. 1, see Supplementary Fig. SS3 for right hemisphere) showed extensive positive relationships across the cortical surface for volume and area. Bidirectional relationships were seen for thickness, especially in development, as expected due to ongoing developmental cortical thinning in this age-range. The volumetric results were most uniform in terms of direction of effects and a broad anatomical distribution. The same analyses were run using each SES variable as predictor, also showing widespread effects with most consistent results for volume (income, see Supplementary Fig. SS4; education, see Supplementary Fig. SS5). This suggests that global GM volume is a good summary measure of brain structure also in relation to GCA and SES. Further GM analyses were thus conducted on global GM volume, hereafter termed GM. Variation in GM and ICV in relation to age are shown in Figure 2 across all cohorts.

### Income and Education

In all main analyses, variables of interest were adjusted for sex and age using a smoothing spline. To compare effect sizes across subsets of cohorts (development vs. adulthood, European vs. US), Wald tests for mean group differences between group-level meta-analytic estimates were run. We ran analyses on the cohorts grouped by age (development vs. adulthood) and in the full sample to obtain meta-analytic effect size estimates of both the grand-average relationships and the group-level relationships. For developmental cohorts, the parental education and income were used as SES.

As income data were given in various bins across studies, it is not possible to provide an informative measure of dispersion of income across studies. However, plots showing the distribution of income in each cohort that had data on income available are depicted in Supplementary Figure SS6. An overview of dispersion of years of education across studies is given in Supplementary Table SS3. A Welch two group *t*-test showed that the variance in education was greater in European than US samples (*t* = 3.9567, df = 3.0835, *P* = 0.0274). The relationship between education and income was significantly positive overall (*r* = 0.30, 95% CI: 0.20–0.40), but heterogeneity was large ($Q$= 2185.99, $P<0.0001$, ${I}^2$ = 99.29%, see Supplementary Fig. SS7). The association was in the positive direction in all 10 cohorts contributing both measures, and reached significance (as evidenced by CI not overlapping 0) for all but 1 cohort (NESDA). While apparently stronger relationships between income and education were found in US (*r* = 0.51, CI: 0.20–.82) versus European (*r* = 0.25, CI: 0.18–0.31) cohorts, the difference was not significant (*Z* = −1.611, *P* = 0.1100).

### Relationships of GCA with SES and Brain Structure

**Figure 3:**
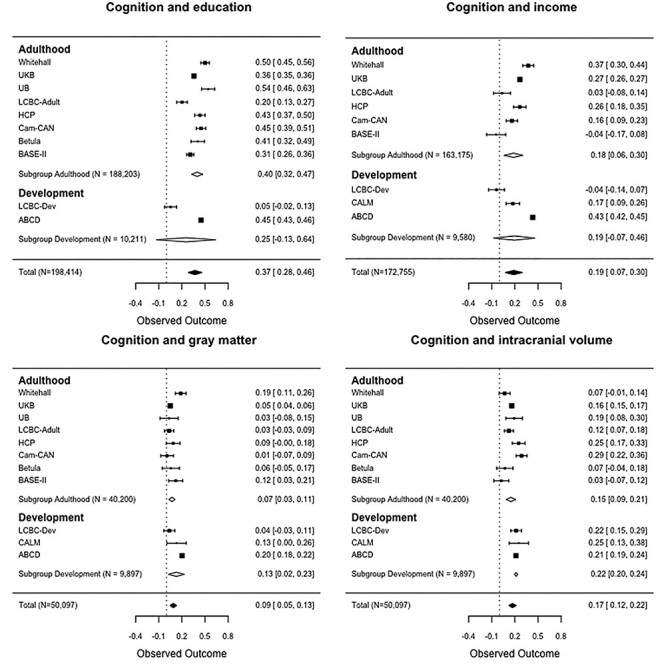
The associations of cognition (GCA) with education, income, GM, and ICV. Forest plots show the individual observed effect sizes with corresponding 95% CI. Diamonds represent the weighted average correlation estimate and its 95% CI with white diamonds representing the subgroup estimates and black diamonds the overall estimate. Numeric values of the cohort-specific and meta-analytic estimates are given in the right column. CI not spanning 0 indicates a significant (*P* < 0.05) relationship.

In analyses with total GM volume ICV was controlled for in addition to the other variables as listed above. Relationships of GCA to education, income, GM and ICV are shown grouped by developmental and adult cohorts in Figure 3. For plots grouped by European versus US, see Supplementary Figure SS8. The overall GCA-education correlation was *r* = 0.37 (CI: 0.28–0.46). The association was significantly positive in 9 of the 10 cohorts, as evidenced by CIs not overlapping 0. However, as the only two developmental cohorts included in this analysis showed very different effect sizes, the association was not significant across the developmental cohorts. The overall GCA-income correlation was *r* = 0.19 (CI: 0.07–0.46). The association was significantly positive in 6 of the 9 cohorts included, but was negative, although not significant, in 2 cohorts (BASE II, LCBC-dev), rendering the associations not significant in development. The overall GCA-GM correlation was *r* = 0.09 (CI: 0.05–0.13). The association was in the positive direction in all 11 cohorts included, but was significantly different from zero in only 4 (Whitehall, UKB, BASE II and ABCD). The overall GCA–ICV correlation was *r* = 0.17 (CI: 0.12–0.22). The association was in the positive direction in all 11 cohorts included, and was significantly different from zero for 8 (Whitehall, Betula and BASE II being the exceptions). Heterogeneity overall was large (GCA-education: ${I}^2$ = 98.93%, $Q$ = 257.91, $P$ < 0.0001; GCA-income: ${I}^2$= 99.05%; $Q$ = 436.60, $P$ < 0.0001; GCA-GM: ${I}^2$ = 89.98%; $Q$ = 194.70, $P$ < 0.0001; GCA–ICV: ${I}^2$ = 92.56%, $Q$ = 63.82, $P$ < 0.0001). The differences between developmental and adult cohorts in the GCA associations did not reach significance (all *P* > 0.25), with the exception of the GCA–ICV association being stronger in the developmental (*r* = 0.17, CI: 0.12–0.22) than adult (*r* = 0.15, CI: 0.09–0.21) cohorts (*Z* = −2.026, *P* = 0.043).

The difference in the GCA-education associations between European (*r* = 0.35, CI: 0.24–0.46) and US cohorts (*r* = 0.45, CI: 0.43–0.46) was not significant (*Z* = −1.672, *P* = 0.095). However, the GCA-income association was significantly stronger in the US (*r* = 0.35, CI: 0.19–0.52) than in European (*r* = 0.14, CI: 0.02–0.25) cohorts (*Z* = −2.09, *P* = 0.037). There was no significant difference in the GCA–GM associations adjusted for ICV between European (*r* = 0.07, CI: 0.03–0.11) and US (*r* = 0.15, CI: 0.04–0.27) cohorts (*Z* = –1.377, *P* = 0.17). However, the GCA–ICV association was stronger for US than European cohorts (European: *r* = 0.15, CI: 0.10–0.21; US: *r* = 0.22, CI: 0.20–0.24; European-US difference: *Z* = –2.055, *P* = 0.04).

### Relationships Between Brain Structure and SES

**Figure 4:**
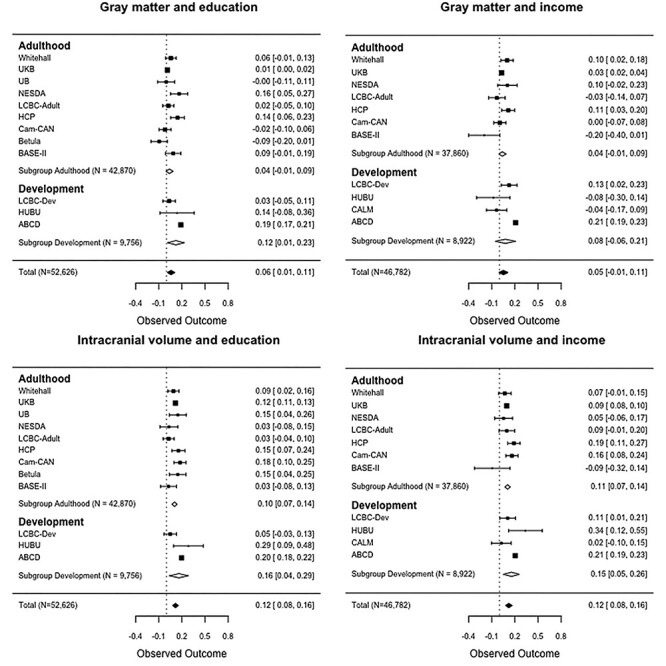
The associations of GM and ICV with education and income. Forest plots show the individual observed effect sizes with corresponding 95% CI. Diamonds represent the weighted average correlation estimate and its 95% CI with white diamonds representing the subgroup estimates and black diamonds the overall estimate. Numeric values of the cohort-specific and meta-analytic estimates are given in the right column.

Relationships of GM and ICV with SES are shown grouped by developmental and adult cohorts in Figure 4. For plots grouped by European vs US, see Supplementary Figure SS9. The overall GM-education correlation was *r* = 0.06 (CI: 0.01–0.11). The association was in the positive direction in 9 of the 12 cohorts included, but only significant in 3 (NESDA, HCP, and ABCD). The effect was in the negative direction, although not significant, in two adult cohorts (Betula and Cam-CAN) and was numerically zero in an additional cohort (UB). There was no overall significant GM-income correlation (*r* = 0.05, CI: −0.01–0.11). The association was in the positive direction in 6 of the 11 cohorts included, and significant in 5 (Whitehall, UKB, HCP, LCBC-Dev, and ABCD), numerically zero in 1 (Cam-CAN), and in the negative direction in 4 cohorts (LCBC-adult, BASE II, HUBU, CALM). There was no significant difference in the GM-income association between developmental and adult cohorts (*Z* = −0.538, *P* = 0.5900). Heterogeneity was large for the GM-education ($Q$ = 248.19, $P<0.0001$, ${I}^2$= 91.74%) and GM-income ($Q$ = 251.14, $P<0.0001$, ${I}^2$=93.56%) associations.

Income was significantly more strongly associated with GM (*Z* = −2.763, *P* = 0.0057) in US (*r* = 0.17, CI: 0.08–0.26) than European cohorts (*r* = 0.03, CI: −0.02–0.07). Education was also significantly more positively related to GM (*Z* = −7.987, *P* < 0.0001) in US (*r* = 0.18, CI: 0.16–0.20) than European cohorts (*r* = 0.03, CI: −0.01–0.06).

The overall ICV-education correlation was *r* = 0.12 (CI: 0.08–0.16). All associations were positive, and significant relationships were observed in 8 of the 12 cohorts included (not in NESDA, LCBC-dev, LCBC-adult, and BASE II). Heterogeneity was large (*Q* = 72.44, $P$ < 0.0001, ${I}^2$ = 85.66%). The ICV-education association was not significantly different (Z = –0.858, *P* > 0.39) between developmental and adult cohorts, but was significantly greater (*Z* = −4.528, *P* < 0.0001) in US (*r* = 0.19, CI: 0.17–0.21) than European (*r* = 0.10, CI: 0.06, 0.14) cohorts. The overall ICV-income correlation was *r* = 0.12 (CI: 0.08–0.16). The associations were positive in 10 of 11 cohorts, being significant in 6 cohorts (UKB, HCP, Cam-CAN, LCBC-dev, HUBU, and ABCD). Heterogeneity was large ($Q$ = 72.44, $P$ < 0.0001, ${I}^2$= 85.66%). The ICV-income association was not significantly different between developmental and adult cohorts (Z = − 0.876, p = 0.3800), but was significantly greater (*Z* = −9.583, *P* < 0.0001*)* in US (*r* = 0.20, CI: 0.18–0.22) than in European (*r* = 0.09, CI: 0.08–0.10) cohorts.

### Sensitivity Analyses

Repetition of the analysis with the samples split by median income, did not show any significant differences in overall correlations between the lower and higher income parts of the samples, for either the relation of income to GM volume controlled for ICV (*P* = 0.8112), ICV (*P* = 0.2082) or GCA (*P* = 0.1768). Repetition of income analyses across US and European cohorts where the lowest 15% income participants were omitted from the US samples, reduced the relationships between income and cognition, GM, and ICV somewhat in the US samples. The difference in the association of cognition and income between US (*r* = 0.29, CI: 0.17–0.40) and European (*r* = 0.14, CI: 0.02–0.25) cohorts was then nonsignificant (*Z* = −1.787, *P* = 0.074). However, the associations of income and GM (US: *r* = 0.14, CI: 0.10–0.17; European: *r* = 0.03, CI: −0.02–0.07; *Z* = −3.671, *P* = 0.0002) and income and ICV (US: *r* = 0.16, CI: 0.14–0.18; European: *r* = 0.09, CI: 0.08–0.10; *Z* = −5.461, *P* < 0.001) were still significantly greater in the US than European cohorts.

Since we found that income and education were more strongly related to ICV than GM and the relationships may thus be grounded in neurodevelopment (see discussion below), we decided to repeat this analysis also without the US ABCD cohort, containing much of the developmental data. These analyses without the ABCD cohort showed the same significant differences, with both education (*P* = 0.0415) and income (*P* < 0.0001) being significantly more related to ICV than to GM adjusted for ICV. For additional details and sensitivity analyses, see section Sensitivity analyses of Supplementary Material.

### Controlling for Ethnicity and Genetic Ancestry

We addressed whether ethnicity or genetic ancestry factors (GAF) affected the relationships. It should be noted in this regard that there is substantial variation across cohorts in ethnicity, and the extent to which ethnic variance is present in the populations from which they were recruited also varies substantially. Controlling for either reported ethnicity (available for UKB, HCP, and ABCD, see Supplementary Figs SS10 and SS11) or GAF (available for UKB, LCBC-adult, and LCBC-Dev, see Supplementary Figs SS12 and SS13) did not change the results substantially in the adult cohorts. In general, also relatively little change was observed in the relationships in the Norwegian developmental cohort (LCBC-Dev) when controlling for GAF, but the ICV-income relationship was no longer significant (GAF-adjusted *r* = 0.04, CI = –0.08–0.15; not GAF-adjusted *r* = 0.11, CI: 0.01–0.21). Relationships in the ABCD cohort appeared attenuated overall, albeit still significant when controlling for reported ethnicity. GCA-education and GCA-income relationships in ABCD appeared stronger when not controlling for ethnicity (going from *r* = 0.45, CI: 0.43–0.46 to *r* = 0.33, CI: = 0.32–0.35 for GCA-education and from *r* = 0.43, CI: 0.42–0.45 to *r* = 0.28, CI: 0.26–0.30 for GCA-income). Associations between GCA and brain measures with SES variables in ABCD were the proportionally most attenuated overall after controlling for ethnicity (going from being in the range of *r* = +/−0.20 to *r* = +/− 0.10), but were still significant.

### The Role of ICV

Differences in SES–CV relationships and SES–GM relationships when ICV is controlled for have implications for the extent to which effects on the brain may be established in development or adulthood/aging. We found that education related more strongly (*P* = 0.039) to ICV (*r* = 0.12, CI: 0.08–0.16) than to GM controlled for ICV (*r* = 0.06, CI: 0.01–0.11). The same was the case for income (*P* = 0.0270) (with ICV: *r* = 0.12, CI: 0.08–0.16; with GM controlled for ICV: *r* = 0.05, CI: −0.01–11). Notably, GCA was significantly more positively related to ICV than to GM controlled for ICV (*P* = 0.0226). GCA was also more positively related to education than to income (*P* < 0.0001). For details on these comparisons, see Supplementary Material, Supplementary Figure SS14.

### Effects of GM and ICV on SES–GCA Relationships

We tested the extent to which the brain variables could explain part of the relations between cognitive ability and SES. We did this by testing the difference between the GCA–SES correlations adjusted for age and sex and the same correlations additionally adjusted for ICV, GM, or both. Overall, the correlations between GCA and SES were larger when not being adjusted for brain variables, especially for ICV. The GCA-education relationship was significantly more positive when not adjusting for ICV (*P* = 0.0014), GM (*P* = 0.0154), or ICV and GM combined (*P* = 0.0160). The GCA-income relationship was also significantly more positive across cohorts when not being adjusted for these brain variables (not adjusting. vs adjusting; for ICV: *P* = 0.0020; GM: *P* = 0.0240; ICV and GM: *P* = 0.0245). There was considerable variance across cohorts in the extent to which these variables altered the correlations. Only in the ABCD, HCP, and the UKB cohorts, however, were SES–GCA associations significantly lower when controlling for any brain variable (see Supplementary Fig. SS15 for details).

## Discussion

In summary, by this multicohort approach we show that there is substantial heterogeneity in SES–brain–cognition relationships across US and European cohorts encompassing all ages of the human lifespan. This demonstrates that SES does not exert influence on either brain or cognition, or vice versa, in any uniform way across cohorts. There were stronger positive relations between SES and brain structure in the US than in the European cohorts. ICV was more strongly related to SES than was GM volume controlled for ICV. This indicates a primarily developmental effect rather than neuroprotection in aging. We also found that ICV and GM volume explained part of the variance in both the education-GCA relationship and the income-GCA relationship.

These results nuance the role of income in brain and cognitive development and aging in cohorts in industrialized countries, as uniformly positive effects were not the rule. As samples are highly heterogeneous and have varying degrees of representativeness of the populations of origin, and lack of population representativity is indeed known (Stamatakis et al. 2021), caution is warranted in interpretation of specificity of effects. While education was as expected related to cognitive ability, and also showed some relationship to GM volume, a stronger relationship was observed for education and ICV.

This may imply that associations between education and brain characteristics are grounded in neurodevelopment, as ICV changes relatively little after school-age is reached, and is known to stabilize between 10 years of age (Pfefferbaum et al. 1994) and midadolescence (Mills et al. 2016). GM volume, on the other hand, for which less effect of education, and no overall effect of income, was found, shows substantial age differences across the lifespan, especially in older age (Walhovd et al. 2005; Walhovd et al. 2011; Fjell et al. 2013). As years of education typically accumulate after ICV no longer increases, a direct effect of education on ICV in adulthood is improbable. Having more educated parents—or a correlate thereof - could have a facilitating effect on brain development in childhood and possibly adolescence. For instance, an association between parental education level and hippocampal volumes was found to be mediated by cortisol levels in children (Merz et al. 2019).

We also note that the relations of SES to brain and cognition were generally of similar magnitude in developmental and adult cohorts, and there were generally not significant differences in the strength of associations with age. This is so despite the fact that we had to apply different measures of SES in developmental and adult cohorts, namely parental versus individual SES.

While individual education often has been seen as boosting development and being neuroprotective (Livingston et al. 2017; Ritchie and Tucker-Drob 2018; Staff et al. 2018), evidence also exists for it being neuroselective (Ericsson et al. 2017; Selzam et al. 2017). Both the boosting development and neuroprotection account implies a causal effect of socioeconomic status, whereas in a strict neuroselective view, education and income would rather be markers or proxies of some other favorable, putatively genetic, trait (Ericsson et al. 2017; Selzam et al. 2017). A higher ICV could in part reflect causal factors in driving years of education in adulthood. However, one needs to keep in mind that ICV has shown very high heritability, up to 0.88 in some studies (Renteria et al. 2014), and genetic pleiotropy of ICV and education may be likely. While for instance education could be a spurious correlate of socially, rather than genetically transmitted advantages, recent evidence points to genetic influences on educational attainment both directly through social mobility and indirectly through family environments (Belsky et al. 2018). The present heterogeneity of SES–brain–cognition associations across cohorts has implications for whether relationships can be assumed to arise from direct or indirect effects of SES in early development or aging. If education really had a neuroprotective effect in aging, then we would expect people with higher education to show less brain atrophy, and hence greater neuroanatomical volumes and also better cognitive function in aging. We saw no evidence that higher SES was specifically related to gray matter volumes and cognitive function in older adulthood. Rather, higher SES could be associated with enhanced maturation, as we generally observed equally strong associations with childhood cognitive function, and stronger relationships to ICV, as a proxy for maximal neuroanatomical volume.

As this study was performed on cross-sectional data, conclusions regarding change in brain and cognition cannot be drawn. Less knowledge exists on SES–brain structure relations in midlife and aging, but a relatively large US study found that community disadvantage in midlife was associated with reduced cortical tissue volume, cortical surface area, and cortical thickness, but not subcortical morphology (Gianaros et al. 2017). Hence, while most focus has been on development, there is no reason to believe that overall relations between SES and brain structure is confined to young samples. Indirect evidence for this also comes from epidemiological data, where lower SES is associated with greater risk of dementing diseases characterized by brain atrophy or lesser neuroanatomical volumes (Livingston et al. 2017). However, the fact that SES–brain–cognition relationships are found in aging cohorts, should not be taken to indicate that they operate in aging specifically, rather than in a stable manner, perhaps as an intercept effect across the lifespan. The current results do not support a neuroprotective account, where higher SES serves to mitigate cognitive decline or GM atrophy in aging. Neither education nor income were consistently positively associated with ICV-adjusted GM volumes, and relationships with GM and cognitive ability did not significantly differ in developmental and aging cohorts.

### Comparison Between European and US Samples

The question of whether SES–brain–cognition relationships differ across cohorts and societies has also been highlighted by other types of studies. Evidence for an SES–genotype interaction on cognitive ability has been found, in terms of suppression of heritability with lower SES (Scarr-Salapatek 1971; Tucker-Drob and Bates 2016). Recently, such effects and as their possible current absence in European and presence in US samples have been debated (Tucker-Drob and Bates 2016; de Zeeuw and Boomsma 2017; Figlio et al. 2017). Our results show substantial heterogeneity of SES–brain–cognition relationships across cohorts also within Europe, and even from the same country, as can be appreciated by differing effect sizes across UK cohorts.

However, there were significantly different effects of income on cognition, and of income and education on GM and ICV between US and European samples. These differences all point to stronger positive relationships between SES and brain and cognition in the US than in the European samples. When the lowest 15% income participants were excluded from the US samples, the association of income and cognition was no longer significantly greater in the US than European samples, whereas the associations of income with GM and ICV were slightly weaker, but remained significantly greater in US than European samples. This indicates that the greater SES–brain-associations in the US cohorts may in part be driven by the lowest income part of the samples, and as income distributions may systematically vary across the presently included samples (see further discussion below), one should not see the differences in associations as intrinsic to US versus Europe. Rather, these findings show that relationships between income, education and brain and cognition found in some large and well-known cohorts should not necessarily be taken to apply across cohorts, regardless of origin. Large US studies on developmental samples, one of which included here, have shown broadly distributed associations between SES and brain structure (Noble et al. 2015; McDermott et al. 2018). One recent European longitudinal study found widespread associations between a composite SES measure and cortical surface area at age 14, with independent contributions from polygenetic scores for education (Judd et al. 2020). Some US studies have found the strongest associations, with especially lower regional neuroanatomical volumes, in children living in poverty (Hanson et al. 2013; Hair et al. 2015). Somewhat less evidence is available from European cohorts, although such associations have also been found in young cohorts in Germany and France with large variation in SES (Jednorog et al. 2012; Holz et al. 2015). However, in a Norwegian sample (Walhovd et al. 2016), including a subset of the one entered in present analyses, no associations were found between income or education and regional cortical area. The current finding of US–European differences is thus not completely unexpected.

However, it should be emphasized that the currently included cohorts will vary in representativeness of the populations from which they were drawn. For both US cohorts, efforts were made to recruit participants reflecting the ethnic and sociodemographic composition of the population (Van Essen et al. 2013; Garavan et al. 2018). Unfortunately, even designing sample demographics to be similar to those of a target nation population, such as in the ABCD, does not guarantee sample representativeness across a multitude of dimensions of interest. For instance, it is known that ABCD under-recruited rural families because of neuroimaging facilities tending to be in mostly urban research centers (Compton et al. 2019). This, we believe, is bound to be the case in European studies too. And while population representativeness was sought also for many of the European cohorts (see e.g., Nilsson et al. 1997; Bertram et al. 2014; Sudlow et al. 2015), this was not necessarily successfully accomplished and some of these cohorts are also in part convenience samples. Thus, differences across US and European cohorts may still reflect more diverse sociodemographic backgrounds in the US than European studies. For instance, the UKB cohort is not representative of the population from which it is drawn with regard to a number of risk factors (Stamatakis et al. 2021). However, it should be noted that in terms of years of education, the variance was greater in European than US samples. As for SES and GCA, a few meta-analyses exist (Bowles et al. 2001; Ng et al. 2005; Strenze 2007), all reporting relationships between intelligence quotient (IQ), income, and education. In the most comprehensive meta-analysis so far, differences in IQ–SES relationships in the USA versus other Western societies were not supported (Strenze 2007). This is in line with our findings for education, but we note that a stronger positive relationship between income and cognitive ability was observed in US samples, though the difference was not significant when excluding the 15% lowest income parts of the US samples. Income was not consistently related to cognitive ability across the present cohorts. The most positive relationships were found in the US and UK cohorts, while there were other European cohorts in which no relationships were seen. Hence, the results highlight that income may not be related to cognitive function in a global way.

### The Role of Brain Structure in SES–Cognition Relationships

Finally, the current results did not only yield support for SES–cognition and SES–brain relationships, but also showed that variance in brain structure, that is, ICV and GM independently of ICV, explained part of the education-cognition and income-cognition relationships across cohorts. This was indicated by the fact that adjusting for either brain metric significantly weakened the relationships. By this analysis, we cannot, and do not intend to say that SES causally affects cognitive function through its effect on brain structure. However, these analyses indicate that partially overlapping variance in brain and cognition is shared with SES as a distant proxy. Future studies, and preferably longitudinal ones, are required to further delineate mechanisms leading to such relationships.

## Limitations and Future Directions

The current study has a number of limitations. Other relations could be uncovered with less general metrics than education, income, GM and ICV. For instance, occupation, subjectively perceived social rank, cortical thickness and area could be more refined measures. Still, the vertex analyses showed that both for GCA and SES, effects were anatomically widespread and more consistently related to volume than thickness or area, suggesting that GM volume is a sensitive measure of brain structure for our purpose. Given that income may be more related to brain and cognition in lower income cohorts (Decker et al. 2020), a skewing of samples towards more wealthy participants may have affected results. While the comparison of correlations between income, brain and cognition in the upper and lower income halves of the samples yielded no significant differences overall, it may still be that consistent relations could be found across samples of lower income. Indeed, excluding the 15% lowest income individuals I the US samples did weaken the income-brain relationships somewhat, and rendered the difference of associations between income and cognition in US versus Europe nonsignificant. Further, ethnicity or GAFs were not included as covariates in all analyses. While some of the cohorts have no or minor ethnic variation, others have more (see Supplementary Material). Analyses in select big cohorts did overall indicate, however, that the relationships in most cases remained significant when controlling for ethnicity or GAFs.

Furthermore, the measures for some of the constructs studied here are quite heterogeneous. Especially, the fact that income was coded differently across studies, so that systematic variance across studies cannot be readily compared, constitutes a major limitation with regard to further interpretation of what the differences mean. Ideally, income measures should also be supplemented by measures of societal services received (e.g., supported child care, housing, schooling etc), so as to yield a fuller picture of income in relation to need. Heterogeneity of measures also apply to estimates of GCA, which were obtained with different tests of crystallized and fluid ability. While behavior tests were taken from the NIH Toolbox (http://www.healthmeasures.net/explore-measurement-systems/nih-toolbox) in the US cohorts, various tests were used in other cohorts. To the extent that the measures used have higher reliability and validity in some cohorts than others, this could lead to higher correlations in those cohorts. This is a possibility that cannot be ruled out. However, we think that the given data sets do not afford an assessment of differential reliability and validity of the measures. The cognitive measures from the NIH toolbox have shown good short term reliability (Weintraub et al. 2013). Digitial batteries such as the NIH toolbox have also been validated against “gold standards” as indicated by tests from Wechsler batteries (Weintraub et al. 2013), and such were used in several of the European cohorts. The variance is however substantial in the European cohorts. While many used paper and pencil test, UKB, for instance used a digital measure of fluid ability that has shown moderate to high reliability (Fawns-Ritchie and Deary 2020). The content and reliability of the GCA measures may not only vary by test versions but also by age. This is however a fundamental problem of cross-cohort analysis that is ultimately unsolvable unless one can prestandardize all measures, and even if this was to be achieved, age differences may remain. In addidtion, if one were to apply one measure of SES consistently across the lifespan, that would need to be parental income and education, rather than individual income and education. Unfortunately, parental SES was only consistently available for the developmental cohorts currently studied. The comparison of European and US cohorts is limited by the fact that we have only two US samples. In part, the same limitation goes for the relatively few developmental relative to adult and aging samples.

As cohorts are not invariably representative of the societies from which they are recruited, and indeed, we know they are not, further interpretation of these possible differences is not warranted here. We want to emphasize that the present study is not designed to delineate the mechanisms underlying differences in SES–brain–cognition associations across cohorts. For instance, this study does not address the effects of poverty specifically. We do not have sufficient cross-cohort information regarding the combination of household income, size and composition, and it would not be clear what criterion for poverty should be applied across cohorts from different nations. In the EU, people are seen as at risk for poverty when they have income below 60% of the national median disposable income, whereas poverty is defined in absolute terms in the USA (Caminada and Martin 2011). Hence, addressing poverty as a mechanism would require other conceptual and empirical analyses. However, the substantial heterogeneity found should prompt researchers to carefully examine relationships before SES indicators are used as covariates of no interest. Such practices may otherwise suppress or inflate variance in relationships of interest in unpredictable ways.

## Conclusion

There is substantial heterogeneity in the relationships of SES to brain and cognition across major European and US cohorts. Based on these results, it is not likely that the effects of SES on cognition are grounded in neuroprotective effects on GM volume in aging. Rather, SES relations established in brain development may be seen through the lifespan. In this regard, stronger relationships of income and education to neuroanatomical volumes were found in US than European cohorts, pointing to SES not signifying the same across different populations from industrialized countries. The present results also indicate that part of the relations between SES and cognition may be explained by variance in brain structure. This does not imply that SES causally affects cognition through its impact on the brain, only that SES as a distant proxy is related to both brain and cognition in partially similar ways. The findings, including the significant heterogeneity of effects across cohorts, have implications for our understanding of whether, when and how SES may impact brain and cognition.

## Supplementary Material

Supplementary_Material_revision2_24062021_bhab248

## Data Availability

The Lifebrain data supporting the results of the current study are available from the PI of each sub-study on request, given appropriate ethical and data protection approvals. Code used for analyses will be shared upon request. Contact information can be obtained from the corresponding author. UK Biobank data requests can be submitted to http://www.ukbiobank.ac.uk and ABCD data requests to https://abcdstudy.org/.

## Notes

A full list of supporters of the ABCD study is available at https://abcdstudy.org/federal-partners.html. A listing of participating sites and a complete listing of the study investigators can be found at https://abcdstudy.org/scientists/workgroups/. ABCD consortium investigators designed and implemented the study and/or provided data but did not necessarily participate in analysis or writing of this report. This manuscript reflects the views of the authors and may not reflect the opinions or views of the NIH or ABCD consortium investigators. The ABCD data repository grows and changes over time. The ABCD data used in this report came from doi: 10.15154/1506087. Part of the research was conducted using the UK Biobank resource under application number 32 048. *Conflicts of Interest*: Christian A. Drevon is a cofounder, stock-owner, board member, and consultant in the contract laboratory Vitas AS, performing personalized analyses of blood biomarkers. None of the other authors have any conflicts of interests to disclose.
